# Structure-Function Relationship of the β-Hairpin of *Thermus thermophilus* HB27 Laccase

**DOI:** 10.3390/ijms26020735

**Published:** 2025-01-16

**Authors:** Beatriz Miranda-Zaragoza, Guillermo A. Huerta-Miranda, Wendy I. García-García, Elizabeth Hernández-Álvarez, Alejandro Solano-Peralta, Jaeyong Lee, Natalie Strynadka, Margarita Miranda-Hernández, Claudia Rodríguez-Almazán

**Affiliations:** 1 Departamento de Micro y Nanotecnologías, Instituto de Ciencias Aplicadas y Tecnología, Universidad Nacional Autónoma de México, Cto. Exterior S/N, C.U., Coyoacán, Ciudad de México C.P. 04510, Mexico; beatriz.miranda@ibt.unam.mx; 2 Instituto de Energías Renovables, Universidad Nacional Autónoma de México, Priv. Xochicalco, Temixco C.P. 62580, Mexico; guillermo.huerta@ibt.unam.mx (G.A.H.-M.); wendy@ier.unam.mx (W.I.G.-G.); mmh@ier.unam.mx (M.M.-H.); 3 Instituto de Geofísica, Universidad Nacional Autónoma de México, Ciudad Universitaria, Ciudad de México C.P. 04510, Mexico; aeliza@geofisica.unam.mx; 4 Facultad de Química, Universidad Nacional Autónoma de México, Ciudad Universitaria, Ciudad de México C.P. 04510, Mexico; asolito@yahoo.com.mx; 5 Department of Biochemistry and Molecular Biology and Centre for Blood Research, The University of British Columbia, Vancouver, BC V6T 1Z3, Canada; jaeyongl@mail.ubc.ca (J.L.); natalie.strynadka@ubc.ca (N.S.)

**Keywords:** laccase, *Thermus thermophilus*, β-hairpin, extremozyme

## Abstract

Thermus thermophilus HB27 laccase (Tth-Lac) is a thermostable enzyme that contains a β-hairpin (Ala292-Gln307) covering the substrate entrance. We analyzed the role of this β-hairpin in the enzymatic activity of Tth-Lac through three β-hairpin mutants: two variants without the β-hairpin (C1Tth-Lac and C2Tth-Lac) and one with a partially modified β-hairpin (P1Tth-Lac). Enzymatic activity was assayed with different substrates with and without copper. C1Tth-Lac showed a higher dependency on copper, increasing its activity by 1600-fold for syringaldazine (SGZ). All mutants presented a higher activity than Tth-Lac with phenolic substrates in the presence of copper. The position of the signal associated with CuT2 also changed, as shown in EPR spectra. Elucidation of the crystal structure of P1Tth-Lac mutant (PDB: 9CPM) showed that the partial deletion of the β-hairpin did not significantly affect the overall tertiary structure compared to the wild-type (PDB: 2xu9) nor the coordination of the four internally bound Cu atoms. Higher B-factors of the residues downstream of the deletion indicate increased flexibility (Q307, G308, P309, S310) that were otherwise more ordered in the Tth-Lac structure. Redox potential experiments on platinum electrodes have shown that all proteins have high redox potential, a finding that could have significant implications in the field of protein research.

## 1. Introduction

The molecules produced by extremophilic organisms have garnered considerable interest in biotechnology applications due to their particular characteristics, such as thermostability [1,2,3]. The metabolism of extremophile organisms employs enzymes with exceptional stability and activity under extreme environmental conditions, leading to the discovery of extremozymes [4]. These have various biotechnological and industrial applications, such as bioremediation, pharmaceuticals, textiles, food and beverage manufacturing, biorefineries, and others [5]. While numerous publications exist on these robust enzymes, characterization is often lacking, primarily due to their low activity in moderate conditions, usually resulting from evolutionary adaptations to extreme environments [2].

The proteins of heat-extremophile organisms are produced in a range often between 50 and >80 °C, with folding optimized through a high prevalence of hydrophobic interactions [4], with additional charged amino acids stabilizing through longer-range electrostatic interactions [6,7]. Furthermore, the presence of short loops in the N- and C-terminal positions helps prevent the unfolding of the protein by avoiding the entry of water to the center of the protein [8,9]. The increase of the rigidity in the protein matrix contributes to a reduction in activity at temperatures < 50 °C but recovers the flexibility of the structure at high temperatures, which leads to favorable catalytic activity under these conditions [10,11].

Usually, to understand the structure-function of a specific site of an extreme enzyme, specific amino acids are replaced by others. However, significant changes in the stability and regulation of the catalytic activity are reflected by changing the overall structure of extreme enzymes or specific domains, such as in loops [12,13]. Loops are irregular regions that form motifs by joining elements of secondary structure; these are classified into five types based on the secondary structure: α-α, α-β, β, β-α, β-β, and β-hairpins [14].

The β-hairpins are motifs composed of adjacent antiparallel β-sheets, stabilized through hydrogen bonds and linked by small loops. These structures are generally located outside the hydrophobic center of the proteins and with both sides exposed to the solvent [15,16]. The presence of β-hairpins has been related to the stability and folding in enzymes that interact with metals, more specifically, copper [15,17]. This motif is present in intein from Archaea and other extremophile organisms, termed an extremophile hairpin (EXH), and participates in stability and protein folding [17]. It has been proposed that in thermo-stable laccases, the β-hairpin and its modification might affect the activity of these enzymes [18].

Laccases are enzymes that belong to the family of multicopper oxidases (MCOs) and catalyze the oxidation of a phenolic and non-phenolic substrate to the reduction of a molecule of oxygen to form two water molecules [19,20]. These enzymes have four catalytic coppers, a type 1 copper (CuT1), close to the surface, and a trinuclear center (TNC) formed by one type 2 copper (CuT2) and two type 3 coppers (CuT3) [20]. Laccases are known for their ability to utilize a wide variety of substrates [21], highlighting their potential for diverse uses in biobleaching processes and bioremediation of xenobiotic compounds in addition to being used to treat textile industry effluents and other industries [22,23].

Bacterial laccases have the greatest thermal stability and a broader catalytic range in terms of pH and temperature [24,25]. Thermus thermophilus laccase (Tth-Lac) is the most thermostable laccase reported at the moment, with an inactivation time of ≈14 h at 80 °C 11 [26,27]. The Tth-Lac is the only laccase that presents a β-hairpin (Ala292–Gln307) close to the substrate binding site and the CuT1 site (Figure S1). The hairpin comprises two antiparallel β-strands (β22 and β23) and a connecting loop of six residues [18,28]. Tth-Lac is a monomer composed of three domains and the only laccase reported so far with the presence of a β-hairpin.

Molecular docking simulations of Tth-Lac with 2,2ázino-bis(3-ethylbenzothiazoline-6-sulfonix aid (ABTS) suggested that the β-hairpin occludes the substrate-binding site [18]. It was proposed that this structure has a motion like a lid, with an open and a closed conformation. Only the laccases from *Campylobacter jejuni*, *Escherichia coli* (CueO), and *Pyrobaculum aerophilum* have structures in similar positions, giving rise to the hypothesis that these structures regulate substrate entry; however, the mechanism and specifics of the role they perform are still unknown [27,29,30]. As an approximation, the deletion of the α-helices occluding the active site showed a thirty-fold increase in the enzymatic activity using as substrate ABTS. On the other hand, the inactivation time (t_1/2_ thermoresistant) measured at 60 °C decreased from 38 min in CueO to 28 min in Δα5–7 CueO; however, the specificity of some substrates increased. The increase in enzymatic activity supports the idea that this structure is an effective barrier to bulky organic substrates and improves access to the type I Cu site, ensuring targeted substrate interaction [19].

Here, we combined biochemical, spectroscopic, electrochemical, and structural assays to determine the participation of the Tth-Lac β-hairpin in the enzymatic activity. Through different Tth-Lac mutants, we found that the composition and length of the β-hairpin impact the number of coppers present, as well as the spectroscopic characteristics of the coppers. The hairpin mutants showed a kinetic behavior like that found in Δα5−7 CueO [19], in which the most significant influence on the activity is related to the phenolic or non-phenolic substrate.

## 2. Results

### 2.1. Construction of β-Hairpin Mutants

**Figure 1:**
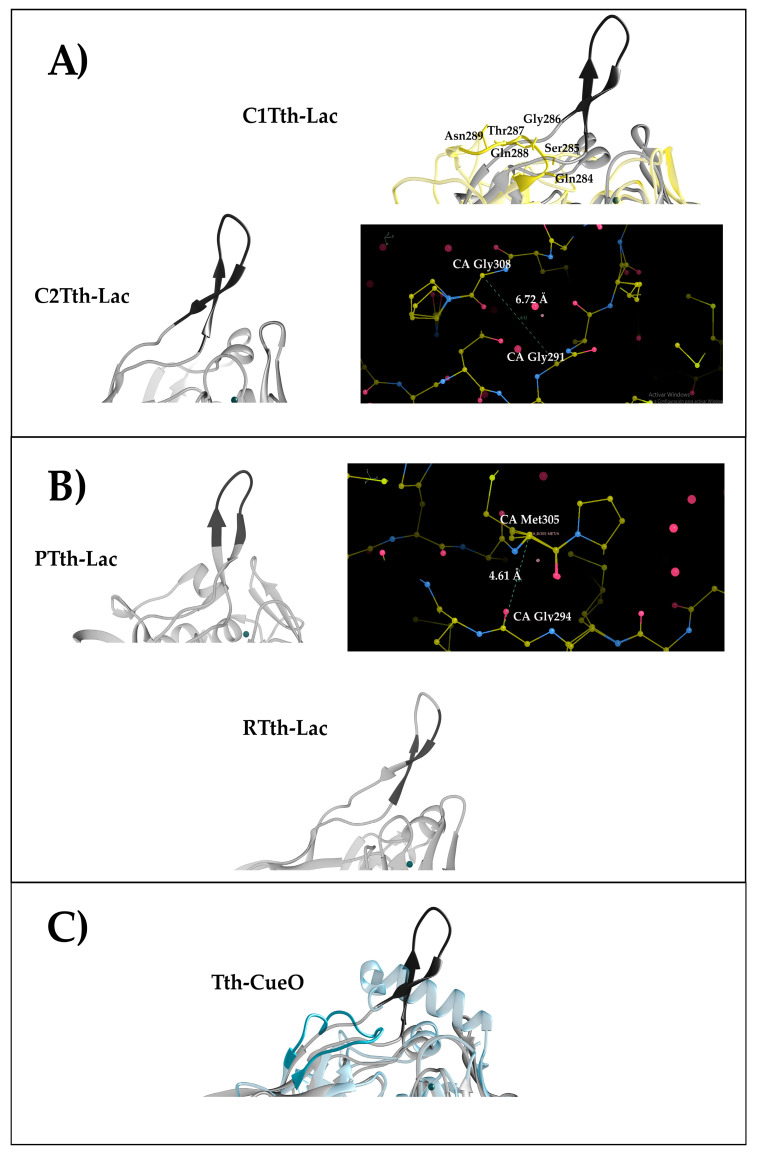
Models of the β-hairpin mutants using Tth-Lac structure (PDB 2XU9). (**A**) Complete deletion of the β-hairpin. C1Tth-Lac was determined from the structural superposition of the Tth-Lac with the laccase from *Coriolopsis gallica* (yellow) (PDB 4A2E), which has no β-hairpin and is the most active laccase reported until now. The atom/bonds representation of C2Tth-Lac shows the distance between the α-carbons of Gly291 and Gly308. (**B**) Partial deletion of the β-hairpin. On the top, P1Tth-Lac and P2Tth-Lac are shown in ribbons and atoms/bonds representations, showing the distance between α-carbons of Gly294 and Met305. On the bottom, the RTth-Lac mutant shows the deleted region. (**C**) Substitution of the β-hairpin with CueO. Structural superposition of the Tth-Lac and CueO (cyan) (1N68). Tth-Lac (2XU9) is in gray ribbon representation. The highlighted regions (dark grey) correspond to deleted residues of the β-hairpin. The atoms/bonds representation shows the distance between the α-carbons of the residues next to the eliminated zone. The model of the mutants were made with SWISS-MODEL, and energy was minimized with YASARA; furthermore, they were analyzed in WinCoot32 and UCSF Chimera.

A β-hairpin element is much more than a connector between other secondary structures. Surface exposure of β-hairpins can play a crucial functional role since they have the potential to interact with ligands and other biomolecules. To explore the role of Tth-Lac β-hairpin in enzymatic activity, we explored the composition and length of the β-hairpin through mutants that consisted of the complete, partial, or site-specific substitution of hairpin residues (Table 1, Figure 1). The molecular construction of β-hairpin mutants was performed using the overlapping technique and directed mutagenesis (Table S1, Figure S2). For all mutants generated, we maintained residues Tyr288 and Arg290 since they have been proposed to be important for substrate binding sites [13].

The mutants C1Tth-Lac and C2Tth-Lac represent the deletion of the β-hairpin with varying linkers substituted. The C1Tth-Lac mutant deletion encompassed the Gly291-Gln307 region with the addition of serine as the linker. This amino acid residue selection was based on the structural overlap of Tth-Lac with *Coriolopsis gallica* laccase, one of the most active laccases reported so far (Figure 1A). In the C2Tth-Lac mutant, the region from Ala292 to Gln307 was deleted, and a Gly residue was added as a connector [31]. Gly residues are often observed in the linking of secondary structural elements since they can enhance flexibility through greater phi/psi freedom [32,33]. The partial mutants were generated to remove eight to ten residues of the β-hairpin (Figure 1A). The deletion of P1Tth-Lac extends from Met295 to His303 with an additional A304G mutation (Figure 1B). P2Tth-Lac was designed using the P1Tth-Lac mutant as a template with an extra Gly as a connector (Figure 1B). In the RTth-Lac mutant, the central region of the β-hairpin was maintained, deleting residues Met293−Met296 and His303−Pro306 (Figure 1C). In the substitution mutant, β-hairpin elements were substituted in entirety by the Tth-CueO loop sequence from *E. coli* laccase (CueO) between residues 291 and 301 (Figure 1C). The CueO loop was selected because it has a similar position to that of the β-hairpin in Tth-Lac 29.

### 2.2. Overexpression and Purification of the β-Hairpin Mutants

**Figure 2:**
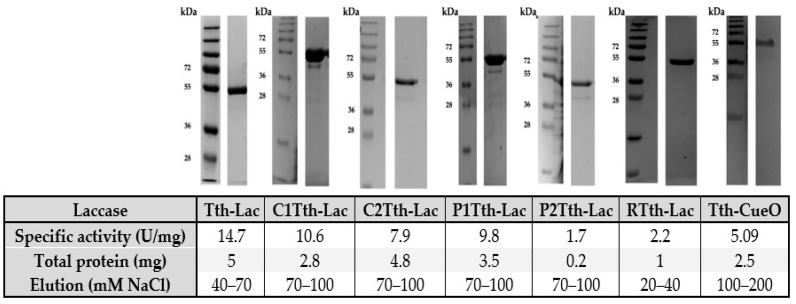
Purified β-hairpin mutants. The top shows the SDS PAGE results for each mutant evaluated. The bottom of the figure shows a table with the specific activity, total protein, and concentration of NaCl. Samples were analyzed on a 12% SDS-PAGE and stained with Coomassie blue brilliant. The activity was assayed at pH 4.5 and 61.5 °C, 20 mM sodium acetate buffer, 1 mM CuSO_4_, and 10 µg of total protein in enzymatic reaction. Protein concentration was determined through the molar extinction coefficient of each mutant. The total purified protein is from 1 L of cell culture. The purification was mainly to heat the extract at 65 °C for 20 min and then in a CM-Sepharose column, buffer 20 mM MOPS, pH 7.4, and a NaCl molar gradient (0−500 mM).

The expression protocol was the same for all mutants. The protocol followed was standardized first for the wild-type enzyme and, when applied to the mutants, provided for a consistent ~90% degree of purity (Figure 2). However, the NaCl concentration for elution, as well as the yield of each protein, was different.

The enzymatic activity and purity by SDS-PAGE gel electrophoresis were evaluated immediately after chromatography. The criteria of selection for the mutants centered on activity, level of degradation, and yield. To determine the storage buffer, all laccases were stored at different pHs (4.5, 5, 5.5, 6, and 7.0) for two weeks and then assayed for enzymatic activity and SDS-PAGE (Figure S2). The mutants C1Tth-Lac, C2Tth-Lac, and P1Tth-Lac showed the best integrity, activity, and yield. According to these results, the three mutants were stored at pH 4.5 and selected to analyze the function of the hairpin in the enzymatic activity of Tth-Lac. The P2Tth-Lac, RTth-Lac, and Tth-CueO mutants had low enzymatic activity and yield compared to the others.

### 2.3. Catalytic Activity in β-Hairpin Mutants

#### The Activity of Laccases at Different pHs

The enzymatic activity of laccases varies according to the substrate, pH of the reaction, temperature, presence or absence of copper in the reaction medium, and other parameters [21,34]. Bacterial laccases work at a wide pH range of 3.0 to 8.0, depending on the specific substrate [22]. The activity was determined using phenolic compounds (DMP, GCL, and SGZ) as phenolic substrates and the non-phenolic compound (ABTS).

**Figure 3:**
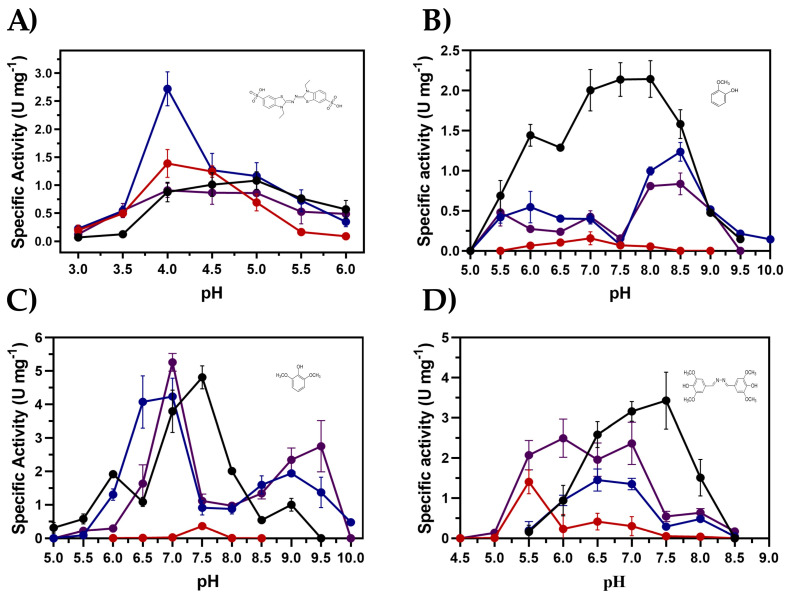
Effect of pH on specific activity using phenolic and non-phenolic substrates. Within each box is the structure of each substrate, in (**A**) non-phenolic substrate ABTS, (**B**) phenolic substrates GCL, (**C**) DMP, and (**D**) SGZ. Tth-Lac is shown in black, C1Tth-Lac in red, C2Tth-Lac in blue, and P1Tth-Lac in purple. In each box, the chemical structure of the specific substrate is represented. The activity was assayed without copper at 61.5 °C, buffer (20 mM), and 10 µg of total protein.

Figure 3A shows the activity–pH behavior of the enzymes with ABTS as substrate. Since the solubility of copper is compromised by alkaline pH, the activity assays were without copper. Tth-Lac exhibited its highest activity at pHs 4.5 and 5.0. The wild-type enzyme was the protein that had activity under more acidic conditions, pH 3.5. In comparison, C1Tth-Lac and C2Tth-Lac showed their highest activity at pH 4.0, between 1.4 and 3 times greater than that of Tth-Lac. Except for C1Tth-Lac, all enzymes have activity at pH 6.0.

Enzymatic activity was assayed with GCL as a substrate (Figure 3B), and laccases showed maximum activity around pH 8.0. except for the C1Tth-Lac. Tth-Lac retained more than 90% activity from 7.0 to 8.0, showing a higher range when compared to the rest.

The behavior of the enzymes with DMP is depicted in Figure 3C. Notably, the wild-type enzyme and C1Tth-Lac displayed their higher activity at pH 7.5. However, the remaining mutants exhibited distinct activity behaviors. C2Tth-Lac and P1Tth-Lac displayed a maximum pH of 7.0. Unexpectedly, only the C2Tth-Lac and P1Tth-Lac maintained activity at pH 9.5.

For the SGZ substrate (Figure 3D), Tth-Lac presented its maximum activity at pH 7.5, while C1Tth-Lac was at pH 5.5 and the rest at pH 7.0. The activity of the wild-type enzyme is 6–10 times greater than that of the rest of the variants at pH 7.5.

The wild-type enzyme consistently demonstrated the highest activity–pH stability relation in all substrates tested, indicating its robust performance over a wider pH range. These findings are significant since they provide valuable insights into the adaptability of this enzyme and its potential applications. The C2Tth-Lac exhibited the highest enzymatic activity for ABTS, while the Tth-Lac excelled for GCL and the P1Tth-Lac for the SGZ and DMP substrates.

The length and composition of hairpins and loops affect the catalytic efficiency and functional conditions in laccases [18,19,35]. Different bacterial laccases have been identified as copper-dependent since their activity is further increased by the addition of this metal [36,37]. To determine the effect of the mutations in the enzymatic activity, we calculated the kinetic parameters of Tth-Lac, C1Tth-Lac, C2Tth-Lac, and P1Tth-Lac using the same four substrates DMP, GCL, SGZ, and ABTS in the presence and absence of free copper.

The addition of copper in the reaction medium allows the laccases to reach their maximum enzymatic activity [38,39]. The activity assays were performed in the presence and absence of copper to obtain the enzymatic parameters. As expected for copper-dependent enzymes, all mutants exhibited a remarkable increase in activity upon the addition of copper (Table 2 and Table 3). The Vmax of Tth-Lac was enhanced twenty-fold with copper, whereas, in the rest of the enzymes, this increase was between five and seven times. C1Tth-Lac had the highest increment in catalytic efficiency, specifically on phenolic substrates, displaying a 1600-fold increase with the GCL substrate. The C1Tth-Lac was the mutant with the highest copper dependence.

The wild-type enzyme displayed the highest activity in all the conditions analyzed, with a few exceptions: C2Tth-Lac and P1Tth-Lac with ABTS substrate and C1Tth-Lac and P1Tth-Lac with GCL (Table 3). The highest catalytic efficiency in SGZ was for Tth-Lac (1850 s^−^^1^ mM^−^^1^) and the C1Tth-Lac mutant (1501 s^−1^ mM^−1^); on the other side, C1Tth-Lac displayed the best affinity (4.83 × 10^−3^ mM). For the substrate DMP, the wild-type exhibited significant catalytic activity (6 U mg^−1^). At the same time, the C2Tth-Lac mutant presented a substrate affinity (Km = 0.22 mM) approximately twice as high as that of the rest of the proteins.

### 2.4. Spectroscopic Characteristics

**Figure 4:**
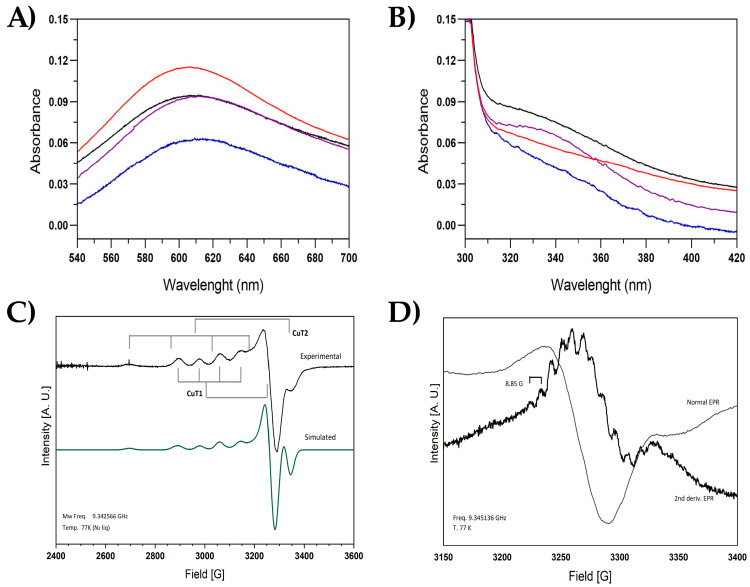
Spectroscopic behavior of catalytic copper atoms of the analyzed laccases. (**A**) CuT1site spectra in UV-Vis. (**B**) CuT3 site spectra in UV-Vis. Tth-Lac is in black, red corresponds to C1Tth-Lac, C2Tth-Lac is blue, and purple represents P1Tth-Lac mutant. (**C**) Experimental and simulated EPR spectra of Tth-Lac at 77 K. (**D**) Second derivative EPR spectrum showing hyperfine coupling to CuT1 site.

The mutants were analyzed using EPR and UV-Vis techniques to evaluate the status of different copper atoms. In all cases, the laccases were expressed with copper but were assayed without free copper. The UV-Vis signal ≈ 610 nm associated with CuT1 was observable for all the laccases analyzed (Figure 4A). However, only the enzymes Tth-Lac and P1Tth-Lac presented the characteristic signal at ≈330 of CuT3 (Figure 4B); this could indicate a change or a loss of CuT3 for C1Tth-Lac and C2-Tth-Lac. The copper content of the laccases was determined by ICP-MS, and the results have shown that only the C2Tth-Lac had 3.83 coppers per protein, while Tth-Lac had 3.28, C1Tth-Lac had 2.13, and P1Tth-Lac had 3.07.

On the other hand, EPR spectroscopy is a useful tool for the study and structural characterization of the Cu(II) metal centers present in the laccase enzyme. The unpaired electron located in the dx2_y2 orbital produces a characteristic S = 1/2 EPR spectrum with g|| > g^ > 2.0023. The two naturally copper isotopes, 63Cu and 65Cu (69 and 31%, respectively), have a nuclear spin I = 3/2, which couples in the parallel component with the unpaired electron, giving a four-line hyperfine splitting of the EPR signal. The CuT1 site exhibits a very small parallel hyperfine splitting [A|| = (43–90) × 10^−4^ cm^−1^] in the EPR spectrum. The trinuclear center, where dioxygen is reduced to water, is comprised of one T2 site with one Cu atom, which exhibits a large parallel hyperfine splitting in the EPR spectra [A|| = (150–201) × 10^−4^ cm^−1^] and a binuclear copper center, CuT3, antiferromagnetically coupled through a bridging ligand and therefore EPR silent (Figure 4C).

In the proteins studied here, all EPR spectra exhibited the anisotropic behavior characteristic of laccases. The spectra comprised two superimposed signals corresponding to CuT1 and CuT2 sites. The spectra show an axial signal with hyperfine splitting in the parallel component. For Tth-Lac, the g-values centered at g|| = 2.210 (A|| = 82.05 × 10^−4^ cm^−1^) and g^ = 2.047 assigned to the CuT1 site and g-values of g|| = 2.251 (A|| = 186.0 × 10^−4^ cm^−1^) and g^ = 2.038 associated to the CuT2 site were obtained (Figure 4C, Table 4). For C2Tth-Lac, the EPR spectrum presented slight differences concerning the rest of the proteins, finding a slight signal shift at higher g values.

When enlarging the perpendicular component of the signal assigned to CuT1 in Tth-Lac, a small super hyperfine splitting, around 3260 Gauss, was observed in the signal, so its second derivate EPR spectrum was obtained and showed in the perpendicular component an eleven-line splitting pattern with an average splitting of 8.85 G (8.46 × 10^−4^ cm^−1^), which was associated to the interaction of the copper electron with the four nitrogen nuclei of Histidine residues in the CuT1 site.

### 2.5. Electrochemical Characterization

The classification of laccases based on the CuT1 redox potential (vs. SHE) exhibits three distinct groups, which is a significant finding. This classification includes low potential laccases (340–490 mV), medium potential (470–710 mV), and high potential (730 mV) [44]. Tth-Lac, as well as the hairpin mutants, were evaluated by cyclic voltammetry (CV) and square wave voltammetry (SWV) adsorbed on platinum electrodes to determine the redox potential value as well as its state of activity under air atmosphere (CVs).

**Figure 5:**
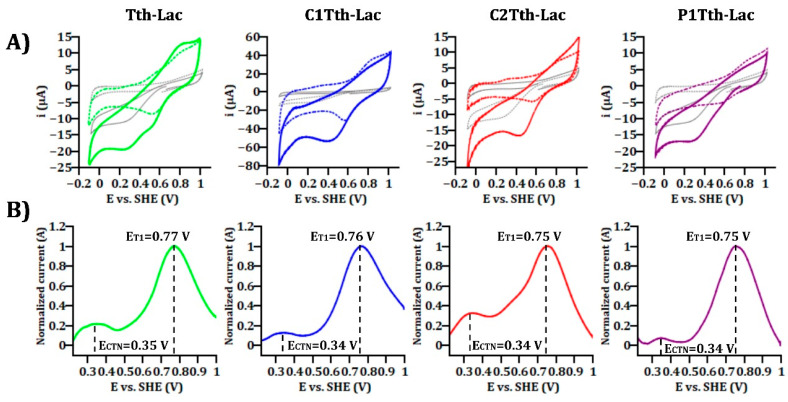
The electrochemical response of the studied enzymes immobilized on Pt electrodes. (**A**) CV responses were obtained at 100 mVs^−1^ in air condition (solid lines) and inert N_2_ atmosphere (dotted lines) and (**B**) SWV responses in air atmosphere. Pt electrode (gray line), Tth-Lac (green line), and the mutants: C1Tth-Lac (blue line), C2Tth-Lac (red line), P1Tth-Lac (purple line).

Figure 5A depicts the electrochemical response of the enzymes in comparison to the bare Pt electrode under nitrogen (dotted lines) and air (solid lines) atmospheres. The adsorbed enzymes exhibited higher current values in both atmospheres than the bare Pt electrode. Despite the electrochemical activity displayed by the bare Pt electrode in the presence of oxygen (gray solid line), the enzymatic systems consistently showed significantly higher current production, as evidenced by the cathodic peak at 0.43 V across all tested enzymatic systems.

Figure 5B shows the normalized SWV responses of the enzymatic systems after subtracting the bare Pt electrode response. The current normalization was based on the peak current of CuT1, denoted as E_T1_ in all cases. Tth-Lac was the protein with the higher redox potential; however, the different hairpin deletions did not significantly affect the redox potentials. The redox potentials of CuT1 in Tth-Lac and its mutants are around 750 mV (E_T1_), indicating that the mutation did not directly influence the oxidative capacity of the metallic center, nor did it significantly affect the CuCTN site, which has a potential value of around 340 mV (E_CTN_); this could indicate that the mutation did not directly influence the oxidative capacity of the metallic center. According to the redox potential calculated for CuT1 from SWV on the platinum electrode, all the laccases analyzed are categorized as proteins with high redox potentials. However, it is essential to note that the electrode material can directly impact these results. Figure S3 shows the responses of modified carbon electrodes with the different laccases and the potential shifts down to 100 mV compared to the platinum electrodes.

### 2.6. Structure of P1Tth-Lac

The protein crystal from the P1Tth-Lac mutant laccase is distinct from those reported for wild-type Tth-Laccase [28,45]. It was grown from different crystal reservoir conditions and at room temperature in a much shorter time. It also has a different unit cell, space group, and lattice contacts (Table S2). The final model at 1.6 Å resolution shows acceptable refinement and stereochemical statistics.

**Figure 6:**
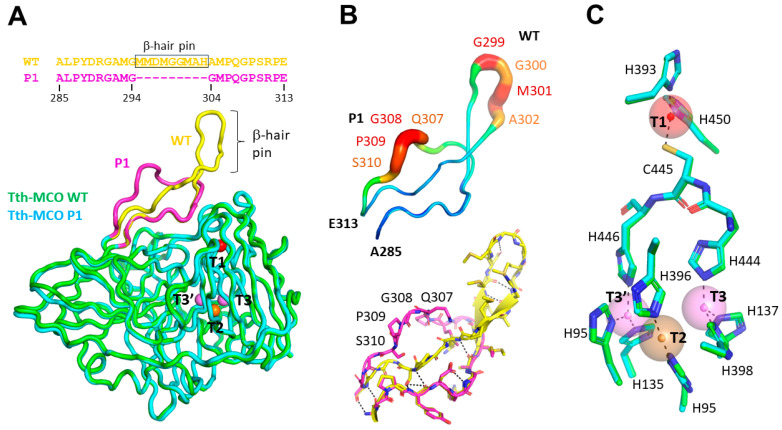
Three-dimensional X-ray crystallographic structure of P1Tth-Lac mutant. (**A**) Cartoon superposition of Tth-Lac (PDB: 2XU9) in green and P1Tth-Lac mutant (PDB: 9CPM) in cyan. The β-hairpin and its flanking sequence (A285-E313) are colored yellow in Tth-Lac and magenta in the P1Tth-Lac mutant. The coordinated copper in P1Tth-Lac mutant is shown in spheres (T1: red, T2: orange, T3/T3′: violet). (**B**) The aligned loop sequences (A285-E313) are shown as B-factor putty (**top**) and as stick form (**bottom**) with stabilizing polar contacts shown as dotted lines in the Tth-Lac structure. (**C**) The aligned Cu coordination residues in Tth-Lac (green) and P1Tth-Lac (cyan). Mononuclear copper (T1Cu) is shown as a red sphere, and T2 and T3/T3′ are shown in orange and violet spheres, respectively.

Compared to the Tth-Lac structure 2XU9 (residues 24–462) [45], the superposition of the refined P1Tth-Lac mutant structure 9CPM (26–462) results in a small rmsd of 0.4817 Å over 421 aligned residues. The majority of mainchain atoms have close superposition with the greatest deviation localized to residues M293 to P312 (Figure 6A). The region with the most structural discrepancy corresponds to the sequence (G304-P312) immediately downstream of the nine deleted residues of the β-hairpin (M295-H303). P1Tth-Lac shows no significant changes in the residues that coordinate the CuT1 site (His393, Cys445, His450, and Met455), nor in the residues proposed to bind the ABTS substrate (Tyr288, Tyr232, Arg290, Met355, Asp390, Met 391, Met354, Lys424).

As would be expected, the shortening of the β-hairpin sequence resulted in the loss of antiparallel beta-strands and the flexible β-loop that covers the substrate channel. But this also appears to have had a knock-on effect on the conformation of the residues downstream. It appears to have destabilized these residues (Q307, G308, P309, S310) and their polar contacts normally present in Tth-Lac. Their high B-factors suggest that they have become conformationally flexible (Figure 6B).

The active site of P1Tth-Lac harbors four copper atoms coordinated by several His residues (Figure 6C). The positions of these sidechains also align well with those in the wild-type Tth-Laccase structure (PDB: 2XU9), which also coordinates four copper atoms in the active site [45].

## 3. Discussion

In the present work, we studied the structure–function relationship of the Tth-Lac β-hairpin through protein engineering, specifically by deleting the β-hairpin and subsequent modifications.

Through catalytic activity assays in a pH range, the pH–activity behavior indicated that the Tth-Lac and mutants were active at higher alkaline pHs and on a broader pH range when compared to those reported for fungal laccases and some bacterial laccases from *B. pumilus*, *B. subtilis* (CotA) [24,38]. Furthermore, C2Tth-Lac and P1Tth-Lac mutants presented activity at pH 9.5, with DMP as substrate, comparable to the value found for LacTT (active at pH 10). Variation in optimal pH could be caused by differences in the ionization and dissociation state [39], causing changes in the pKa of the residues responsible for the recognition and interaction with the substrate.

The activity assay, conducted with free copper in the reaction mix, revealed a surprising increase in enzymatic activity regardless of whether a phenolic or non-phenolic substrate was used. The increase ranged between twenty and thirty times, with the only exception being C1Tth-Lac, which displayed an increment of more than 1600-fold. This unexpected result challenges existing knowledge and stimulates further investigation. Despite the structural similarity between C1Tth-Lac and C2Tth-Lac, C1Tth-Lac appears to lose its conformation or catalytic copper due to the change of two residues. C1Tth-Lac was the variant with the lowest amount of copper per molecule and the lowest catalytic in conditions without free copper. This behavior could be caused by the affection of the substrate recognition site (at ≈ 5 Å). Although these changes are ≈ 10 Å from CuT1, it has been described that the β-hairpin is very mobile and could affect the second coordination sphere [18]. The difference in activity and copper dependency has led us to hypothesize that the β-hairpin deletions might be causing changes in the coordination geometry or the number of coppers. It has been described that serine mutations in cooper coordination sites can be conducive to the loss of these metals [46]. The addition of this metal allows its reincorporation into the laccase structure, as observed in other bacterial laccases [22,47]. We propose that the changes in Km are a result of a larger laccase population that contains all the catalytic coppers and the correct conformation in substrate recognition.

As shown in Table 3, the calculated kinetic parameters showed apparent differences in all substrates. Even though the residues in the binding site were not modified, the affinity and activity significantly differed between the mutants and the Tth-Lac. The changes in kinetic parameters may be due to a modification in the ionization state of residues responsible for substrate recognition. A two-fold or higher increment of the activity for GCL was observed for all laccases, suggesting that the activity can benefit from increasing the interactions in the substrate area. In all the laccases analyzed, the affinity for phenolic substrates was two orders of magnitude higher than that of fungal and bacterial laccases reported [48,49,50,51].The Km values of the mutants were between 4 and 50 mM for GCL and SGZ, displaying similar or higher affinity than other bacterial laccases [48,51]. Despite the structural similarity between DMP and GCL, the affinity and activity for DMP are very low compared to reports [50,51].

In order to determine the presence of coppers, we measured the ratios at absorbance 280/610 and 280/330, which indicate the proportion of protein/CuT1 and protein/CuT3, respectively (Table S3). In the laccases tested, the ratios displayed approximately half of those reported for other laccases [52,53,54]. The CuT3 center could not be visualized by UV-Vis spectroscopy in the laccases. C2Tth-Lac was the only enzyme with four catalytic coppers; in contrast, C1Tth-Lac has only two. Even though C1Tth-Lac and C2Tth-Lac are almost identical, the difference between these mutants may be due to the distortion in the copper coordination site caused by the inclusion of serine in C1Tth-Lac. There are reports about laccases, such as *C. gallica*, CotA, and *Botritys aclada*, quickly losing some of the catalytic coppers in the reaction and the crystallographic structure. CuT2 is reported to be the copper site with the lowest occupation in the crystallographic structures and is proposed to be the most labile [40,54,55]. These findings significantly impact our understanding of laccase enzymes and their structural characteristics, sparking further exploration.

The analysis of the EPR spectra showed that all of the enzymes studied here differ from the values reported for bacterial laccases [41,56]. In the Tth-Lac and mutants studied, the g_||_ and g_⊥_ values were kept constant, except for the C2Tth-Lac mutant. This mutant slightly differed in the CuT2 parameters (g_||_ = 2.455, g_⊥_ = 1.998). Fluctuations in the values of the coupling constants do not necessarily indicate a loss or substitution of ligands and coppers; they could reflect changes in the geometry of metal centers as a consequence of variations of non-coordinating residues [42,55,57]. Additionally, when compared with the simulation of EPR spectra (Figure 4), all proteins showed a change in the position of the signal associated with CuT2. The increased accessibility to the substrate may allow the entrance of a more significant amount of solvent, which could distort a trigonal geometry due to the new polar interactions, as described for CotA [55]. Constant A increase may suggest a change in the bond between copper and its ligands, with a rise in binding distance and distorting in the copper geometry. The changes in g suggest a distortion in the symmetry of the vicinity of CuT2. These differences correspond to a shift in the magnetic environment, probably in the coordination of CuT2. The CuT2 site binds the hydroxyl intermediate during catalysis [58]; alterations in the copper geometry could reflect changes in Vmax.

The electrochemical techniques allow the characterization of the redox properties of an enzyme. In the literature, numerous studies exist on the immobilization of laccases on electrodes made from various materials. Notably, electrodes based on carbon materials allow the observation of oxygen electroreduction catalyzed by the enzyme without the need for a mediator in a precise and efficient way [59,60]. Figure 5A shows a reduction current proportional to the oxygen concentration in these materials, and it is associated with the direct charge transfer between the electrode and the enzyme-catalyzed reaction. The biological nature of the enzyme influences the potential at which the oxygen reduction current is observed [59,61].

The electrode material also plays an essential role in the electrochemical characterization of laccases and other enzymes. When the electrode material is gold, under anaerobic conditions (absence of oxygen), two reversible electrochemical processes can be distinguished, each corresponding to the copper sites. Various authors conclude that the process at higher redox potential corresponds to a fraction of the laccase molecules oriented through the CuT1 site, while the process at lower redox potential corresponds to another fraction of the enzymes oriented through the CuCTN site [62]. In the voltammetric response of laccase from the fungus *Trametes hirsuta* correctly coupled to a bulk gold electrode under anaerobic conditions, as reported by [63], the two processes correspond to the charge transfer of the copper sites facing the electrode. The CuT1 site corresponds to the process at more positive potentials of 0.9 V (vs. SHE). There are few studies in the literature where both copper sites appear in the voltammetric responses, as the proper orientation of the enzyme is needed. Reports about electrostatic interactions between the electrode and the enzyme modulate orientation towards one site or another. Hitaishi (2018) [64] reported that by modifying a bulk gold electrode with different proportions of organic molecules, such as 6-mercaptohexanoic acid and 4-aminothiophenol, a bilirubin oxidase (an enzyme from the laccase family) could be directed preferentially towards one Cu site or the other.

In our results, the CV graphics (Figure 5A) showed only one redox process, including all the mutants; due to the orientation of the enzymes, one of the sites favored Pt electrodes. However, the primary purpose of the CV studies was to verify qualitatively the modification of the electrodes. As observed in Figure 5A, the modified Pt electrodes presented different responses from the ones of the bare Pt electrode, including higher current generation in the air atmosphere than Pt. However, the intrinsic capacitance of the CV technique did not allow for the determination of the redox potentials associated with the copper sites of the enzymatic systems.

SWV was employed to avoid the capacitive current, enabling the direct determination of E°’ (the formal potential) of the enzymatic redox centers [65,66]. In Figure 5B, peaks associated with CuT1 and CuCTN are distinctly visible. These peaks, completely free from the influence of the current produced by the bare electrode, are solely associated with the enzymes. Compared to the rest of the bacterial laccases, Tth-Lac and the hairpin mutants showed higher redox potentials than previously reported [12,13,14,15,16]. The electrode material influenced the potential values. Figure S3 displays the SWV of the enzymatic systems immobilized on carbon electrodes. The carbon electrode without enzymes exhibits a redox process at 0.43 V. The plots of the enzymatic systems show no influence of the carbon electrode on the current, indicating that the enzymes drive the observed processes. For all enzymatic systems on carbon electrodes, the potential for CuT1 hovers around 670 mV (typical of medium potential laccases), while the potential for CuCTN is approximately 230 mV. These results imply that the redox potential of the enzymes remains consistent when transferring electrons to a current collector despite the mutations. Therefore, differences in enzymatic activity are possibly due to other factors.

The change in the coordination of metal atoms observed between the Tth-Lac and the C2Tth mutant, as detected by EPR but not by electrochemical methods, may be attributed to the differing sensitivities of these techniques. EPR spectroscopy offers insight into the interaction between copper atoms in the enzyme’s active site at the atomic level [67,68]. In contrast, electrochemical methods primarily assess the enzyme’s catalytic activity and kinetic behavior, influenced by the overall response of the chemical environment [69]. The factors contributing to changes in the redox potential and activity in laccases are influenced by diverse conditions, including the stability and geometry of CuT1, the interaction network, and the hydrophobicity of the substrate binding site (18–21). Eliminating the β-hairpin from Tth-Lac through various mutants resulted in increased enzymatic activity despite no modifications to the proposed substrate-binding residues; however, removal of this structure expanded the area of the interaction, likely altering the environment of the CuT1 site.

As secondary structures, loops have a significant role in the function and structure of proteins since they are elements that interact immediately with the environment surrounding the proteins, including various molecules. Modifying loops in proteins can alter ligand binding, and this, in turn, protein conformation [70]. In the case of Tth-Lac, the P1Tth-Lac mutant was the most unstable and least active mutant when compared to other mutants. From this stand-alone structure, it is difficult to derive the reason for its low stability and activity conclusively. It shows the coordination of all four Cu atoms by their coordinating residues, and they align well with the Tth-Lac structure, suggesting there was no disruption of the tertiary structure or the active center. The deletion had minimal effect even on the CuT1 site, which is closer to the loop.

Tth-Lac is an attractive enzyme with high application potential. The findings suggest that engineering the β-hairpin could produce more effective enzymes for various applications in biotechnology, particularly in bioremediation and industrial processes.

## 4. Methods

### 4.1. Reagents

New England Biolabs provided the restriction enzymes used. Agilent’s QuickChange Multi Site-Directed kit was used for single mutations. pET-22b (+) and *E. coli* BL21 DE3 (GOLD) strains were donated by Dr. Paul Gaytan Colín (IBt-UNAM). The reagents used to prepare the culture media (MCD LAB, Escobedo, Mexico) and SDS-PAGE gels (all reagents and electrophoresis system were from Bio-Rad, Hercules, CA, USA), as well as the substrates and buffers, were purchased from Sigma-Aldrich, St. Louis, MO, USA.

### 4.2. Construction of Hairpin Mutants

The mutant models were analyzed in WinCoot32 [71] and UCSF Chimera [72]. Tth-Lac structure (PDB 2XU9) gene is ≈1.4 kb, starts at residue 23 (UniProt TT_C1370), and is in the plasmid pET-22b (+). The assembly of the mutant C1Tth-Lac, P1Tth-Lac, Tth-CueO, and RTth-Lac consisted of two rounds of PCR in a T100 Thermal Cycler, Bio-Rad, and the products obtained from the first round were used as the forward and reverse oligonucleotides for the second round of PCR. The oligonucleotides used for the ends in all cases were FwN5′ (Fw 5′-AAGGAGATATACATATGCAAGGC-3′), which encodes the N-terminal region of the protein, and RvE3′, which encodes the C-terminal (5′-CAAGCTTGTCGACGGAGCT-3′). Reactions were carried out with Vent DNA polymerase: 5 min at 95 °C, 30 cycles (95 °C for 30 s, Ta for 30 s (Table S2), 72° for 30 s), and extension at 72 °C for 10 min.

Both PCR products and the vector were digested with NdeI and EcoRI restriction enzymes, using Cutsmart buffer 1× (New England Biolabs, Ipswich, MA, USA); the reaction was assayed for 2.5 h at 37 °C. The product obtained was desalted using amicons with a cut-off of 3 kDa. The sample was centrifuged at 11,384 × *g* for 30 min at 4 °C, washed with MilliQ water, and the sample was concentrated to a final volume of 30 μL. Ligation was carried out following a 1:5 vector–insert molar ratio, 0.25 U/μL T4 ligase, 50 μM ATP, at 18 °C for 14 h. The product was verified by sequencing and used to transform electrocompetent E. coli MC 1061 ΔTrpF cells. The C2Tth-Lac and P2Tth-Lac mutants were obtained by point mutations using the Quick Change Multi site-directed kit, taking the laccases, C1Tth-Lac and P1Tth-Lac, respectively, as templates. The kit protocol was followed for 1 min at 95 °C, 30 cycles (95 °C for 1 min, 55 °C for 1 min, and Ta for 2.8 min). To eliminate the parental DNA, the product obtained was digested with the enzyme DpnI for 1 h at 37 °C; subsequently, it was desalted using amicon, following the previously described procedure. The DNA was used to transform *E. coli* MC 1061 ΔTrpF cells. Finally, purified DNA was extracted, and the construct was sequence verified.

### 4.3. Overexpression and Purification

Protein overexpression was conducted in *E. coli* BL21 Gold (DE3) cells. Cultures were incubated in LB medium with ampicillin (200 mg mL^−1^) at 37 °C and 150 rpm. Induction was carefully timed at OD_600_ = 0.8, using isopropyl-β-D-1-thiogalactopyranoside (IPTG) and 1 mM CuSO_4_. After induction, we reduced the temperature to 30 °C in agitation for 4 h. After the stated time, we stopped the agitation and maintained the culture for 12 h to achieve a microaeration environment of 57.83. Cells were collected by centrifuging at 4000× *g* for 30 min at 4 °C. Cells were resuspended in lysis buffer (50 mM MOPS, pH 7.4), with PMSF as a protease inhibitor. Cell disruption was achieved in a Cole-Palmer ultra homogenizer on ice. The extract was heated at a precise 65 °C in a water bath for 25 min and clarified by centrifugation, 35,000× *g* for 20 min at 4 °C. The supernatant was purified on the CM-Sepharose column (Fast Flow, GE). This column was equilibrated with five column volumes of 20 mM MOPS, pH 7.4. The protein was eluted using a NaCl stepped gradient. We collected and analyzed fractions with 12% SDS-PAGE electrophoresis gels and stained them with Coomassie brilliant blue.

### 4.4. Catalytic Behavior

The enzymatic activity of laccase was determined with the substrates 2 2′-azino-bis(3-ethylbenzothiazoline-6-sulfonic acid) (ABTS) 1 mM (*ε*_420_ = 36,000 M^−1^ cm^−1^), guaiacol 1.5 mM (GCL) (*ε*_465_ = 12,100 M^−1^ cm^−1^), 2,6 dimethoxyphenol 4 mM (DMP) (*ε*_468_ = 49,600 M^−1^ cm^−1^), and syringaldazine 50 mM (SGZ) (*ε*_525_ = 65,000 M^−1^ cm^−1^). We evaluated the activity-pH relationship in the pH ranges 3.0–6.0, 5.5–9.0, 6.0–9.5, and 5.5–8.5 to ABTS, GCL, DMP, and SGZ, respectively. The kinetic parameters were measured in the absence and presence of free cooper. The absence condition consisted of ABTS (0.1–2.0 mM), pH 4.5; GCL (0.02–1.5 mM), pH 6.0; DMP (0.08–4 mM), pH 7.0; SGZ (2.0–100 mM), pH 7.5. On the other hand, the copper addition, 1 mM CuSO_4_, was tested with the different substrates at a specific pH of 6.0.

Different buffers were used according to the pH of the analysis: Gly-HCl at pHs 3.0–3.5; acetate at pHs 4.0–5.5; MES in pH 6.0–6.5 range; MOPS at pHs 7.0–7.5, Tricine at pH 8.0–8.5, and CHES for pHs 9.0–10.0. The reactions were performed at 1 mg mL^−^^1^ and 10 mg mL^−1^ of protein for conditions in the presence and absence of copper, respectively. The parameters were obtained by analyzing two different lots; each measurement was made in triplicate. Spectrophotometric measurements were performed for UV-vis spectra at 61.5 °C using a Cary 60 spectrophotometer, and the temperature was regulated using a Peltier system from Agilent. The parameters were calculated by fitting a nonlinear Michaelis-Menten model using the OriginPro 2019 software.

### 4.5. Spectroscopic Characterization

The presence of all types of copper was evaluated by determining its UV-vis and EPR spectra. The UV-Vis spectrum was obtained by measuring a range of wavelengths 700–250 nm at a scanning rate of 1 nm s^−1^ in a UV-Vis spectrophotometer (Agilent, Hong Kong) at room temperature. Laccases were dialyzed in 20 mM acetate, pH 6.0 buffer.

The EPR spectra were measured in a Bruker Elexys E500 at X-band (9.3 GHz) and 100 kHz modulation. Proteins were used at ≈167 pM in 20 mM phosphate buffer, pH 7.0. The samples were loaded in capillary tubes, frozen with liquid nitrogen, and read at 77 K. The equipment was maintained with a continuous nitrogen flow. For the simulation of the EPR spectrum, we used the easyspin 5.2 in Matlab.

### 4.6. Copper Determination

The copper quantification assay was performed using induced coupled plasma mass spectroscopy (ICP-MS) and the iCAP Qc equipment (Thermo Scientific, Waltham, MA, USA). The samples, 22–47 mmol, were digested by adding HNO_3_ in a two-step process. The assay was carried out in an UltraWAVE (Milestone, Brondby, Denmark) digestor, a first step of 15 min and the second of 10 min, both at 220 °C, 110 bar, and 1500 W. A calibration curve of 0–100 µg L^−1^ was performed. The instrumental error was corrected with the Indio internal standard (In of 10 µg L^−1^).

### 4.7. Redox Potential Determination by CV and SWV

In order to favor the orientation of the copper sites towards the electrode, the formation of a covalent bond of the enzyme with the electrode material was promoted through the modification of the Toray-type conductive carbon paper electrode (FuelCellStore SKU 590637, FuelCellStore SKU 590637). The modification of the electrodes and the characterization assays were carried out in a three-electrode cell, using Ag/AgCl as a reference electrode and a platinum mesh as an auxiliary electrode, with an Autolab PGSTAT 30 potentiostat.

The electrodes were mounted on a non-conductive rigid base. To allow manipulation of the setup, we left a 0.5 cm^2^ working surface exposed. To modify the electrode, we applied potential sweeps using the cyclic voltammetry (CV) technique. Electroreduction was carried out in an aqueous solution of diazonium salt (aminobenzoic acid) to promote the formation of the aryl-carboxylic compound on the carbon paper electrode; this functional group immobilizes the laccases studied by forming an amide bond. This stage was carried out by applying six CV cycles in 10 mM NaNO_3_ and 10 mM 4-aminobenzoic acid.

The enzymes were incubated on the modified electrodes at a concentration of 3.2 mg mL^−1^ for 24 h at 4 °C, maintaining a 1:10 ratio of the proteins to the binding agent. ECD/NHS (N-ethyl-N’-(3-dimethylamino) propyl carbodiimide/N-hydroxy succinimide) (50:50) were used as binding agents.

The enzymes Tth-Lac, C1Tth-Lac, C2Tth-Lac, and P1Tth-Lac were evaluated by cyclic voltammetry (CV) and square wave voltammetry (SWV), both performed in 20 mM sodium acetate buffer, pH 5 with 1 mM KNO_3_, under two conditions: nitrogen and air atmosphere. The potential window range was −0.3 to 0.8 mV at a scan rate of 10 mV s^−1^. Three to five cycles of power scans were performed in the aforementioned window.

### 4.8. Crystallization, Structure Determination, and Refinement

The P1Tth-Lac mutant in 20 mM sodium acetate buffer, pH 6, and 1 mM CuSO_4_ was concentrated to 20 mg mL^−1^ and screened for crystallization using the sitting drop vapor diffusion method. The crystallization condition that resulted in the best diffracting crystal was 0.1 M MES (pH 6.5), 12% PEG 20 000 at room temperature. The crystal was transferred to a cryo condition, which was the same crystal condition supplemented with 30% glycerol, and was briefly incubated before freezing in liquid N_2_ for X-ray diffraction.

The data were collected at the Canadian Light Source beamline CMCF-ID using a tuned wavelength of 0.95371 Å. The data processing was carried out by Dials [73] and subsequent steps were carried out using the CCP4 package [74]. Data reduction was performed by Aimless [75]. The molecular replacement search using the protein chain of Tth-MCO (PDB: 2XU9) was performed with Phaser [76]. The model building and refinement were carried out by COOT [71] and Refmac [77], respectively. Data statistics are provided in Table S2.

## Figures and Tables

**Table 1:** Amino acid sequences of the Tth-Lac β-hairpin in wild-type and mutant forms. The residues of Tth-Lac replaced are blue, while the dashes represent the eliminated amino acid residues, and the amino acid added is in red.

Deletion	Laccase	β-Hairpin Sequence
**None**	**Tth-Lac**	RG ** AMGMMDMGGMAHAMP ** QGP
**Complete**	**C1Tth-Lac**	R ** S ** ----------------GP
	**C2Tth-Lac**	RG ** G ** --------------- ** G ** P
**Partial**	**P1Tth-Lac**	RGAMG--------- ** G ** MPQGP
	**P2Tth-Lac**	RGAMG-------- ** GG ** MPQGP
	**RTth-Lac**	RGA----DMGGMA----QGP
**Substitution**	**Tth-CueO**	** RMGMAIAPFDKP **

**Table 2:** Kinetic parameters of laccases are assayed without copper in the reaction medium at pH 6.0 and 61.5 °C. Vmax (μmol mg^−1^min^−1^), Km (mM), and kcat (s^−1^).

Laccase	ABTS			SGZ			GCL		
	V_max_	K_m_	k_cat_/K_m_	V_max_	K_m_	k_cat_/K_m_	V_max_	K_m_	k_cat_/K_m_
**Tth-Lac**	28.48 ± 1.93	1.03 ± 0.16	22.94	13.92 ± 0.31	6.17 × 10^−3^ ± 0.50 × 10^−3^	1850.77	5.14 ± 0.11	0.021 ± 0.002	205.72
**C1Tth-Lac**	12.34 ± 0.91	0.28 ± 0.06	35.61	14.60 ± 0.80	7.83 × 10^−3^ ± 1.35 × 10^−3^	1501.26	9.47 ± 0.46	0.045 ± 0.008	168.51
**C2Tth-Lac**	14.80 ± 1.43	0.94 ± 0.19	12.82	7.20 ± 0.46	7.20 × 10^−3^ ± 1.13 × 10^−3^	803.26	3.98 ± 0.21	0.037 ± 0.005	86.96
**P1Tth-Lac**	21.48 ± 1.81	1.09 ± 0.16	15.96	6.17 ± 0.85	21.18 × 10^−3^ ± 6.89 × 10^−3^	251.81	9.57 ± 0.47	0.0499 ± 0.006	155.14

**Table 3:** Kinetic parameters of laccases measured with copper in the reaction medium. The reaction was carried out at 61.5 °C, pH 4.5 for ABTS, pH 6.0 for GCL, pH 7.0 for DMP, and pH 7.5 for SGZ. Vmax (μmol mg^−1^ min^−1^), Km (mM), and kcat (s^−1^).

Laccase	ABTS			SGZ			GCL			2,6 DMP		
	V_max_	K_m_	k_cat_/K_m_	V_max_	K_m_	k_cat_/K_m_	V_max_	K_m_	k_cat_/K_m_	V_max_	K_m_	k_cat_/K_m_
**Tth-Lac**	1.44 ± 0.054	0.30 ± 0.04	3.98	2.29 ± 0.21	0.73 ± 0.13	2.61	4.68 ± 0.32	18.67 × 10^−3^ ± 2.58 × 10^−3^	207.37	6.00 ± 0.24	0.88 ± 0.08	5.58
**C1Tth-Lac**	1.71 ± 0.17	0.79 ± 0.19	1.78	0.16 ± 0.04	1.44 ± 0.49	0.1	0.05 ± 0.002	4.82 × 10^−3^ ± 0.67 × 10^−3^	8.18	1.45 ± 0.66	6.96 ± 3.87	0.21
**C2Tth-Lac**	2.97 ± 0.48	1.47 ± 0.44	1.7	0.60 ± 0.05	0.35 ± 0.06	1.4	1.40 ± 0.08	10.63 × 10^−3^ ±1.21 × 10^−3^	104.72	0.87 ± 0.4	0.22 ± 0.03	3.2
**P1Tth-Lac**	2.96 ± 0.49	2.85 ± 0.80	0.88	0.35 ± 0.02	0.24 ± 0.03	1.19	2.75 ± 0.40	36.85 × 10^−3^ ± 9.75 × 10^−3^	62.68	1.45 ± 0.07	0.43 ± 0.06	2.75

**Table 4:** EPR parameters of some laccase.

Laccase	CuT1				CuT2				Ref.
	g_||_	g_⊥_	A_||_ (10^−4^ cm^−1^)	A_⊥_ (10^−4^ cm^−1^)	g_||_	g_⊥_	A_||_ (10^−4^ cm^−1^)	A_⊥_ (10^−4^ cm^−1^)	
**Tth-lac**	2.21	2.05	82.05	8.46	2.25	2.04	186		This work
**C1Tth-lac**	2.2	2.05	84.75	8.26	2.24	2.04	184		This work
**C2Tth-lac**	2.21	2.05	84.75	8.22	2.26	2.03	159		This work
**P1Tth-lac**	2.21	2.05	81.05	7.96	2.25	2.04	186		This work
***Meiothermus ruber*** **DSM 1279**	2.22		82		2.24		192		[40]
**Parental CotA**	2.23	2.04	70		2.26	2.04	183.4		[40]
***CotA-XynA***	2.05	2.04	70		2.25	2.06	183.4		[41]
***CotA-XynAG3***	2.05	2.04	70		2.25	2.06	183.4		[41]
***B. amyloliquefaciens***	2.22		88.2		2.27		181.2		[42]
***Resting oxidized Slac***	2.22	2.04	84.88	9.25	2.21	2.05	198.48	27.47	[43]

## Data Availability

The original contributions presented in this study are included in the article/Supplementary Materials. Further inquiries can be directed to the corresponding author.

## Supplementary Materials

The following supporting information can be downloaded at https://www.mdpi.com/article/10.3390/ijms26020735/s1.
