# Supplementary material for: Structure-Function Relationship of the β-Hairpin of Thermus thermophilus HB27 Laccase
Source: Int J Mol Sci. 2025 Jan 16;26(2):735. doi: 10.3390/ijms26020735 (PMC11766367; doi:10.3390/ijms26020735)
Supplement: Supplementary file 1 [file ijms-26-00735-s001.zip › ijms-3379640-supplementary.pdf]

SUPPLEMENTARY MATERIALS

Structure-function relationship of the β-hairpin of *Thermus thermophilus* HB27 laccase

Beatriz Miranda-Zaragoza, Guillermo A. Huerta Miranda, Wendy I. García-García, Elizabeth Hernández Álvarez, Alejandro Solano-Peralta, Jaeyong Lee, Natalie Strynadka, Margarita Miranda-Hernández, Claudia Rodríguez-Almazán.

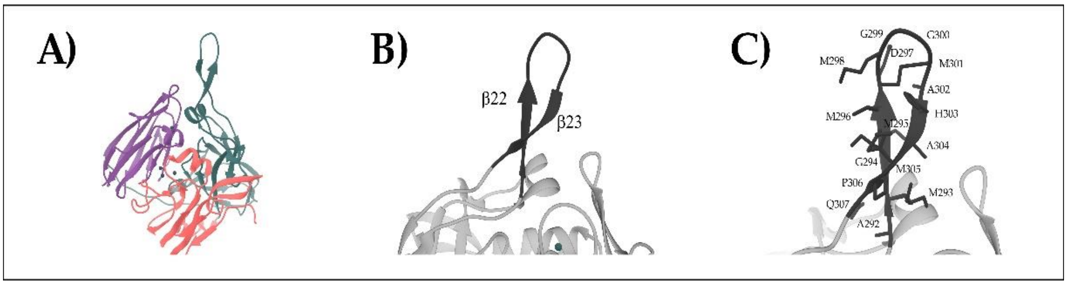

Figure S1. Three-dimensional structure of TthLac (PDB 2XU9). A. Different domains of Tth-Lac, where domain 1 (Gly24-Ala169) is pink, domain 2 (Glu170-Val343) is blue, domain 3 (Val344-Gly462) is purple, and the cooper sites are dark blue dots. B. Beta hairpin and CuT1 copper. C. Residues that make up the beta hairpin.

Table S1. Oligonucleotides used in the construction of Tth-Lac β- hairpin mutants

Protein	Oligo	Sequence (5'-3')	T_a (°C)
Tth-Lac	FwN5'	AAGGAGATATACATATGCAAGGC	55
	Rv C3'	CAAGCTTGTCGACGGAGCT	
C1Tth-Lac	Fw	CAGGCGCTGCCGTATGATAGCGGCCCGAGCCGTCCGGAAACCCTGCTG	55
	Rv	CAGCAGGGTTTCCGGACGGCTCGGTCCACTATCATACGGCAGCGCCTGCAG	
C2Tth-Lac	Fw	TGCCCTACGACCGCGGTGGGGGTCCAAGCCGGCCCGAAAC	59
P1Tth-Lac	Fw	CTGCCCTACGACCGCGGGGCCATGGGCGGGATGCCCCAAGGGCCAAG	53.5
	Rv	CTTGGCCCTTGGGGCATCCCGCCCATGGCCCCGCGGTCGTAGGGCAG	
P2Tth-Lac	Fw	TACGACCGCGGTGCCATGGGAGGAGGAATGCCCCAAGGGCCAAG	58.2
RTth-Lac	Fw1	GACATGGGAGGCATGGCCCACGCAA	51.6
	Rv1	GGCTCCGCGGTCGTAGGGCAGGGCCT	
	Fw2	CAAGGACCAAGCCGGCCCGAAA	59
	Rv2	GGCCATGCCCACCATGTCTGCCCCGCGGT	
Tth-Cueo	Fw	TCCAGGCCCTGCCCTACGACCGCATGGGGATGGCGATTGCGCCGTTTGATAAG	59
	Rv	TCCATTTCTTCCCAAAGCCCGAATAGTTTGCCGCGTTAGCGGTAGGGGTAC	

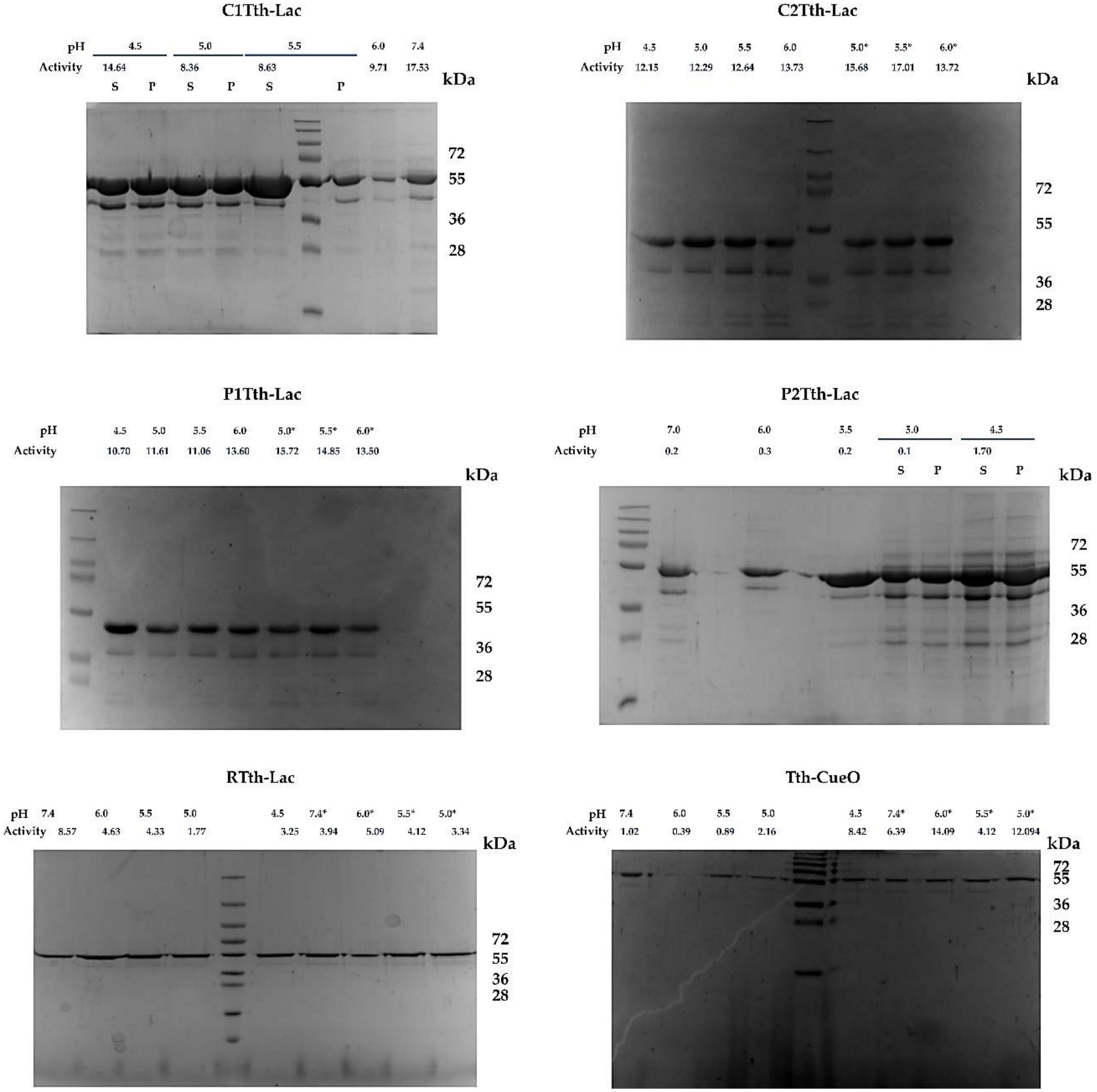

Figure S2. Storage of the β-hairpin mutants. Evaluation of the storage at different pHs: 7.4, 6.0, 5.5, 5.0 and 4.5. The asterisk (*) in the pHs indicates that two dialysis steps were required. SDS-PAGE (12% w/v acrylamide), Coomassie blue staining. All the units for the activity calculations are in U mg^{-1}.

Modification of carbon electrodes for Tth-Lac and mutant enzymes immobilization

To favor the orientation of the copper sites towards the electrode, the modification of the Toray-type conductive carbon paper electrode (FuelCellStore SKU 590637) promoted the formation of a covalent bond of the enzyme with the electrode material.

The electrodes were mounted on a non-conductive rigid base. To allow manipulation of the setup, we left a 0.5 cm^2 working surface exposed. To modify the electrode, we applied potential sweeps using the cyclic voltammetry (CV) technique. Electroreduction was carried out in an aqueous solution of diazonium salt (aminobenzoic acid) to promote the formation of the aryl-carboxylic compound on the carbon paper electrode; this functional group immobilizes the laccases studied by forming an amide bond. This stage was carried out by applying six CV cycles in 10 mM $NaNO_3$ and 10 mM 4-aminobenzoic acid.

The enzymes were incubated on the modified electrodes at a concentration of 3.2 mg mL^{-1} for 24 h at 4°C, maintaining a 1:10 ratio of the proteins to the binding agent. ECD/NHS (N-ethyl-N′-(3-dimethylamino) propyl cardodiimine/N-hydroxysuccinamide) (50:50) were used as binding agents.

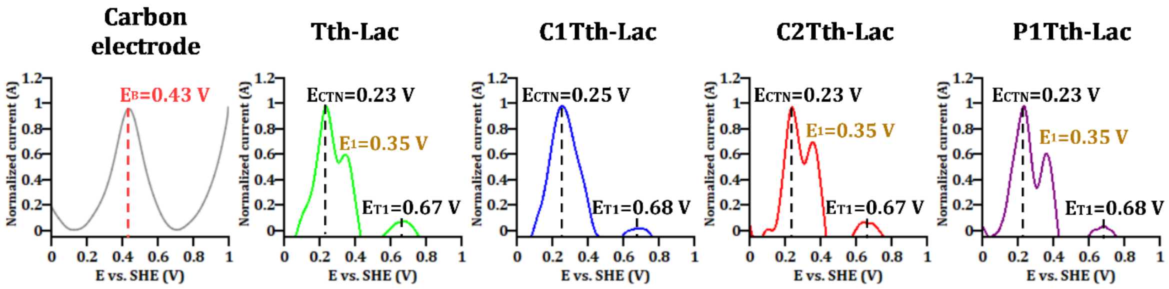

Figure S3. SWV responses in air atmosphere. The modified carbon electrode (gray line), Tth-Lac (green line), and the mutants: C1Tth-Lac (blue line), C2Tth-Lac (red line), and P1Tth-Lac (purple line). The current normalization was based on the peak current of CuCTN, denoted as E_{CTN} in all cases.

Table S2. X-ray data collection and refinement statistics.

**Data collection statistics**	
Wavelength (Å)	0.95371
Space group	P 1 21 1
Unit cell dimension	
a, b, c (Å)	51.93 66.03 74.06
a, b, g (deg)	90.00 100.14 90.00
Resolution range (Å)	66.03 – 1.60 (1.63 – 1.60)
Rmerge	0.184 (2.330)
Rpim	0.116 (1.515)
Total no of observations	448376 (21403)
Total number unique	65047 (3216)
Mean <(I)/d (I)>	7.2 (1.7)
CC(1/2)	0.993 (0.464)
Completeness	99.9 (99.8)
Multiplicity	6.9 (6.7)
**Refinement statistics**	
Resolution range (Å)	51.17 – 1.60
Rwork (Rfree)	0.1535 (0.1815)
Monomers per AU	1
No. of atoms	
Protein	3372
Water	477
Copper ions	5
Average B	17.8
RMS deviations	
Bond length (Å)	0.0117
Bond angles (°)	1.8527
Ramachandran plot	
Favored (%)	315 (90.3)
Allowed (%)	33 (9.5)
Outlier (%)	1 (0.3)
PDB code	**9CPM**

Table S3. Protein-copper rates at 280/610 and 280/330 for CuT1 y CuT3 respectively.

Lacasse	$A_{280/610}$	$A_{280/330}$
Tth-Lac	10.13	10.72
C1Tth-Lac	8.88	N.D.
C2Tth-Lac	16.47	N.D.
P1Tth-Lac	10.89	13.65
